# Supplementary material for: The genome sequence of a cranefly, Diogma glabrata (Meigen, 1818)
Source: Wellcome Open Res. 2025 Jan 15;10:20. [Version 1] doi: 10.12688/wellcomeopenres.23462.1 (PMC11868750; doi:10.12688/wellcomeopenres.23462.1)
Supplement: Peer review history

# Reviewer report: Reviewer response for version 1

**DOI:** 10.21956/wellcomeopenres.25872.r117751

**Published:** 2025-02-27

**Authors:** Marcus Stensmyr

**Affiliations:** 1 Lund University, Lund, Sweden

The manuscript presents the genome of *Diogma glabrata, *a cranefly . The genome has been sequenced as part of the Darwin Tree of Life (DTOL) project, and comprises 1328MB assembled into 4 "chromosomes", as well as the mitochondrial genome. The genome sequencing and assembly follows the standard DTOL state-of-the-art pipeline. The genome is, however, not annotated, which makes this resource less useful. Ideally the authors should run the standard Ensembl pipeline (like most other DTOL papers use).  The genome of an additional cranefly genome is a welcome addition to the field of Diptera genomics.

Are sufficient details of methods and materials provided to allow replication by others?

Yes

Is the rationale for creating the dataset(s) clearly described?

Yes

Are the datasets clearly presented in a useable and accessible format?

Yes

Are the protocols appropriate and is the work technically sound?

Yes

Reviewer Expertise:

diptera genomics

I confirm that I have read this submission and believe that I have an appropriate level of expertise to confirm that it is of an acceptable scientific standard.

# Reviewer report: Reviewer response for version 1

**DOI:** 10.21956/wellcomeopenres.25872.r118057

**Published:** 2025-02-18

**Authors:** Saverio Brogna

**Affiliations:** 1 University of Birmingham, Birmingham, England, UK

This note reports the sequencing of the genome of an adult female specimen of the insect *Diogma glabrata* that was collected from Wytham Woods, near Oxford. The summary statistics demonstrate a satisfactory quality of the sequencing and assembly.

 Although the DNA sequencing is of satisfactory quality and therefore useful, it is unfortunate that the poly(A) RNA was possibly not sequenced alongside, as this would have been helpful in decoding the genome.

 It is not clear why the specimen was dissected or what the purpose of this was. If any sample material remains, it would be useful to extract and sequence the poly(A) RNA or to provide an explanation for why this was not done

Are sufficient details of methods and materials provided to allow replication by others?

Yes

Is the rationale for creating the dataset(s) clearly described?

Yes

Are the datasets clearly presented in a useable and accessible format?

Yes

Are the protocols appropriate and is the work technically sound?

Yes

Reviewer Expertise:

Eukaryotic gene expression and RNA processing with an interest in molecular revolution.

I confirm that I have read this submission and believe that I have an appropriate level of expertise to confirm that it is of an acceptable scientific standard.
